# Review—Recent Advances in FSCV Detection of Neurochemicals via Waveform and Carbon Microelectrode Modification

**DOI:** 10.1149/1945-7111/ac0064

**Published:** 2021-05-20

**Authors:** Harmain Rafi, Alexander G. Zestos

**Affiliations:** 1 Department of Chemistry, American University, Washington, DC 20016, United States of America; 2 Center for Neuroscience and Behavior, American University, Washington, DC 20016, United States of America

## Abstract

Fast scan cyclic voltammetry (FSCV) is an analytical technique that was first developed over 30 years ago. Since then, it has been extensively used to detect dopamine using carbon fiber microelectrodes (CFMEs). More recently, electrode modifications and waveform refinement have enabled the detection of a wider variety of neurochemicals including nucleosides such as adenosine and guanosine, neurotransmitter metabolites of dopamine, and neuropeptides such as enkephalin. These alterations have facilitated the selectivity of certain biomolecules over others to enhance the measurement of the analyte of interest while excluding interferants. In this review, we detail these modifications and how specializing CFME sensors allows neuro-analytical researchers to develop tools to understand the neurochemistry of the brain in disease states and provide groundwork for translational work in clinical settings.

Many human diseases and disorders can be traced back to an imbalance of biomolecules and neurotransmitters (NT). Although we can identify which are implicated for certain disease states, detection, differentiation, and quantification is still a challenge for analytical chemists and neuroscientists alike. Electrochemical techniques have been used to resolve this issue, but not all methods are equal in successfully detecting neurochemicals. Amperometry involves the application of a constant potential to monitor the electron transfer of an analyte.^1^ Larger molecules, such as cholesterol have been detected using amperometry with enzymatic surface modification.^1,2^ Additionally, neurotransmitters such as dopamine^3^ have also been detected using amperometry charging currents are not generated because of the constant held potential. However, although amperometry provides high sensitivity for a single molecule, it has relatively low selectivity compared to techniques such as voltammetry.^3^ Other electrochemical detection methods include electrochemical impedance spectroscopy or EIS. EIS measures the resistive and capacitive properties of an analyte by applying a periodic voltage signal. Impedimetric detection has been used to observe immunological binding between an antibody and an antigen where minute changes in impedance are proportional to concentration of the antigen.^4^ Additionally, this detection method has been utilized for DNA, protein, and cell apoptosis sensors.^5^ The EIS working electrode can be easily modified for high specificity in detection, however, the identical conditions and surface regeneration are not easily reproducible.^5^ False positive results must be analyzed with precaution, especially with the low signal to noise ratio.^7^

In addition to these electrochemical techniques, quantifying neurotransmitters has been accomplished using sampling techniques such as microdialysis. Used in tandem with liquid chromatography and mass spectroscopy (LC-MS), this technique allows for multiplexed detection of neurotransmitters.^6,130^ Since many analytes, such as dopamine, are not CNS (central nervous system) permeant, microdialysis requires in situ measurements. A cannula is implanted into a specific area of the brain with a buffer solution, such as Ringers, perfusing through a microdialysis probe. The probe membrane has a semi-permeable sheath to allow for the passage of small molecules, rather than larger macromolecules. Samples are collected and quantified using high performance liquid chromatography through columns that are selective for the analyte of choice.^7^ This method allows for freely behaving animal studies and pairing locomotor activity with dopamine measurements.^8^ However, it does not have fast subsecond temporal resolution at the rate of neuronal firing. The size of the probe also makes targeting specific brain areas difficult leading to poor spatial resolution in addition to immune response induced tissue damage.^9^ Although these and other methods exist, they lack the spatiotemporal resolution that voltametric sensors such as CFMEs have.

Dopamine (DA) is a catecholamine and a chemical messenger in the central nervous system and is found in brain regions such as the substantia nigra and basal ganglia. DA is synthesized by dopaminergic neurons, stored in the neuron’s vesicles, and released to influence the reward-motivation behavior.^11,12^ Dopamine, and serotonin, an indolamine NT, are also implicated in major depressive disorder (MDD), which affects nearly 4.5% of all adults in the United States.^13^ In MDD, the mechanism of action of the serotonin (5-HT) is not fully understood. It is also debatable if only the depletion of 5-HT is the sole cause for clinical depression.^14^ These are just two of the many biologically relevant molecules where enhanced detection and quantification can aide in furthering our understanding of the roles of these NTs in diseases. FSCV and carbon fiber microelectrodes (CFMEs) match the time scale of neurotransmitter release with their combined high temporal and spatial resolution. Utilizing rapid scan rates produces cyclic voltammogram (CV), a current versus voltage plot.^15^ The CV acts as a “fingerprint” where the shape and the position of the voltammogram peaks are specific for each neurochemical. A waveform is applied onto the surface of electrode which encompasses the reduction and oxidation potentials of the molecule of interest. The frequency and scan rate can be adjusted to optimize spatial and temporal resolution, respectively.^16^ Although FSCV has been used in the past, recent advances have led to substantial progress in detecting many biologically relevant molecules beyond DA and 5-HT. We begin by detailing recent advances made in waveform optimization for analyte detection in addition to how CFME surface modification can enhance detection by improving selectivity and decreasing electrode fouling. In this review we detail recent advances made in the analytical technique, fast scan cyclic voltammetry (FSCV), for detection of neurotransmitters with the use of carbon fiber microelectrodes. Rapid measurements of transient neurotransmitter release in vivo will aide in better understanding human diseases like schizophrenia and Parkinson’s, the second most common neurodegenerative disease.^10^

## Dopamine and carbon electrodes.—

CFMEs are individually made by aspirating a ~7-micron carbon fiber into a 1.2 mm glass capillary tube. Commonly used fibers include Goodfellow USA and Cytec Thornel. Using a pipette puller, the glass capillary is stretched to a tapered point and is sealed using oven-cured epoxy resin. The protocol has been used numerous times in the fabrication of CFMEs which adds to consistency and reproducibility between numerous research labs.^17^ Through electrochemical pretreatment, the surface area and roughness of the electrode increases the amount of carbonyl and hydroxyl functional groups on the fiber’s surface and is constantly renewed by an oxidative etching process.^18^ A silver-silver chloride (Ag/AgCl) disk or pellet is used as a reference electrode that is submerged in the same buffer solution washing over the CFME. The CFMEs, or the working electrode’s potential is observed with respect to the fixed Ag/AgCl reference potential of 0.197 V.^19^ Typical buffers used are phosphate buffered saline (PBS), Tris buffer, or artificial cerebrospinal fluid (aCSF). Although they are inherently salt solutions to mimic physiological pH, differences in ionic strength can impact the resulting sensitivity signal.^20^

**Figure 1.**
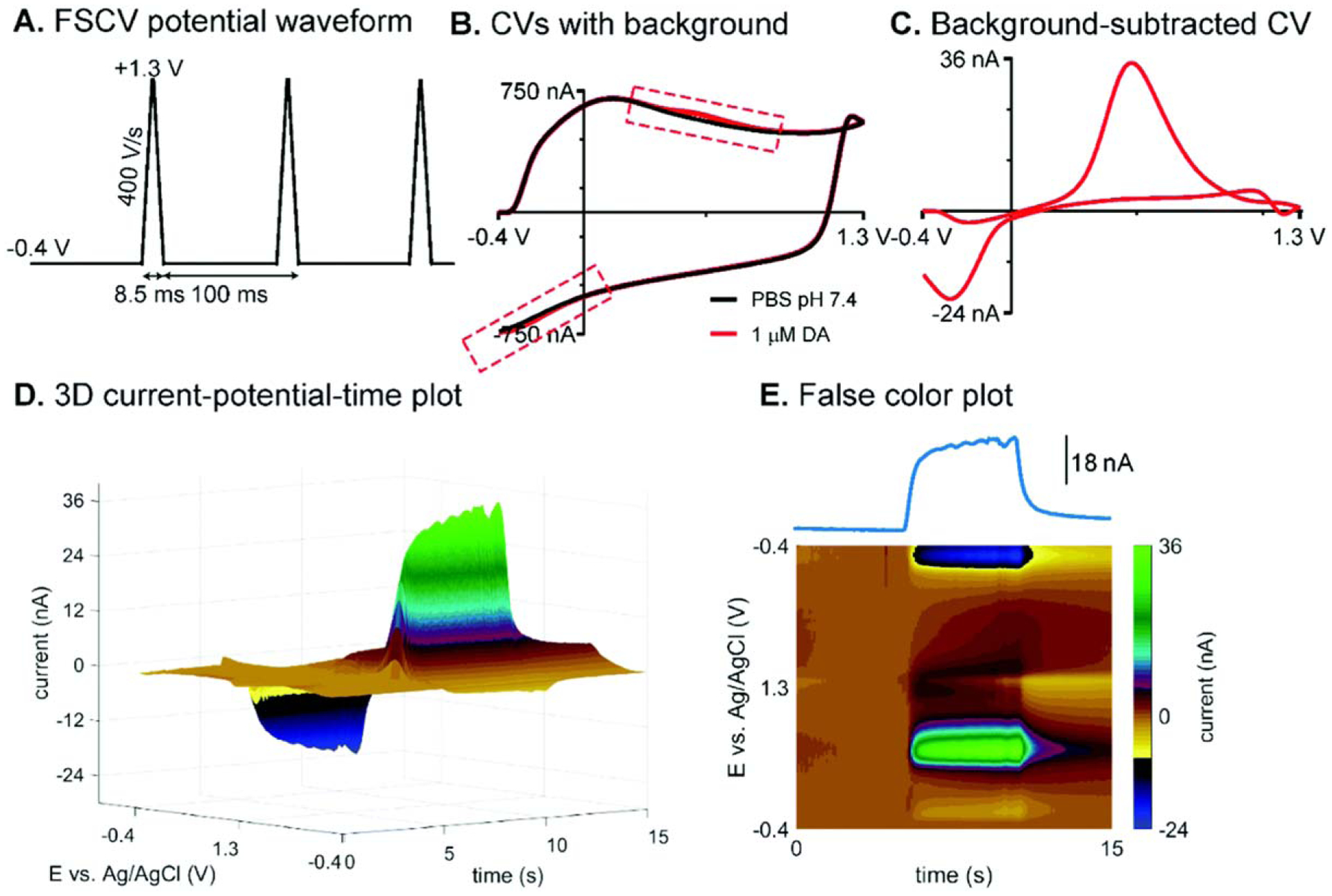
The detection of dopamine with FSCV and CFMEs. (A) Applied potential waveform using −0.4 V holding potential, +1.3 V switching potential, 400 V s^−1^ scan rate, and 10 Hz repetition rate. (B) Example CVs with background: blank (PBS pH 7.4) (black) and buffer with 1 *μ*M dopamine (red). Dashed boxes emphasize the difference between them. (C) Background-subtracted CV of 1 *μ*M dopamine. (D) Three-dimensional current–potential–time plot and (E) conventional false color plot with anodic peak current–time trace of 5 s bolus injection of 1 *μ*M dopamine. Reproduced from Ref. 24 with permission from The Royal Society of Chemistry. (Puthongkham and B. J. Venton, Analyst, 2020, 145, 1087 DOI: 10.1039/C9AN01925A).

To detect dopamine, the common triangle waveform is applied onto CFMEs. The triangle waveform scans from the holding potential (lower limit) of −0.4 V to a switching potential (upper limit) 1.3 V at 400 V s^−1 18^ (Fig. 1A). Utilizing a flow cell (Pine Instruments, Durham, NC), the CFME can be lowered into a well or opening where a constant stream of buffer flows past the exposed carbon fiber. The flow cell has tubing junctions which allow for injections of analytes. When the triangle waveform is applied, using commercially available software, such as HDCV (UNC, Chapel Hill, NC), WCCV (Knowmad Technologies, Tucson, AZ), and Demon (Wake Forest Baptist Medical Center, Winston-Salem, NC) among others, a CV is produced (Fig. 1B). The fast scan rate is necessary to ensure DA kinetics can be observed where DA is oxidized at approximately + 0.7 V on the forward scan to dopamine-o-quinone and reduced back to DA at approximately −0.2 V on the backward scan.^18,21^ Dopamine typically oxidizes around 0.6 V when scanning at 400 V s^−1^. A consequence of scanning at such high rates is the creation of a large non-faradaic background charging current which increase proportionally to the scan rate. However, because charging currents are similar in each experimental trial per electrode, they can be subtracted out to divulge a faradaic current, also known as a background subtracted CV (Fig. 1C).^22^ The oxidation and reduction of an analyte can also be seen in false color plots (Fig. 1E), while taking time into account. The waveform voltage is shown on the *y*-axis, and time is displayed on the *x*-axis, while color indicates current.^23^ The yellow to navy blue hues represent negative reduction current, whereas the green hues represent positive, oxidative currents. The color plot can also be reproduced as a 3D plot which shows the change in potential and current at each time point (Fig. 1D).^24^ Higher switching potentials can over-oxidize the surface of the electrode and enhance sensitivity by breaking carbon-carbon bonds to increase surface area and roughness,^25^ which is a key property for faster DA adsorption/desorption.^26^ Furthermore, it can functionalize the carbon electrode with negatively charged oxide groups, such as carboxyl groups, to electrostatically attract cationic neurotransmitters such as dopamine and serotonin.

In addition to in vitro detection, DA has been successfully measured at a fast, subsecond timescale in animal studies of addiction. In a rodent model, a CFME was inserted into the rat nucleus accumbens (NAc), a dopamine rich area of the brain.^27^ Freely moving rats were allowed to self-administer cocaine, via a catheter implanted into the jugular, every time they pressed a lever. It was found that extracellular DA levels rose just before the lever press and immediately after, showing the changes in DA levels in real time using FSCV with CFMEs.^27^ A similar response was also observed with cannabinoid activation of another dopaminergic area, the ventral tegmental area (VTA) which projects to the NAc.^28^ Increased firing of DA neurons in short bursts have been related to sensory processing related to reward and lead to increased concentration of DA.^29^ Cannabinoid receptor agonists also increased DA levels in these areas.^30,31–33^ These small bursts of DA signal were measured CFMEs provided the high temporal resolution needed for in vivo kinetics of dopamine.^28^ Understanding the dynamics of DA in addiction and receptor binding is imperative to visualizing pathology of addiction diseases.

The triangle waveform parameters and resulting peak positions are specific to DA when using a conventional CFMEs. The same waveform may not be ideal for every ionizable analyte or for simultaneous co-detection. Waveform development has helped create assays for enhancing the detection of neurotransmitters. By altering the scan rate, analytes with slower electron transfer kinetics can be detected and differentiated from other molecules. Moreover, specific neurotransmitters are measured by specialized waveforms to enhance detection including providing a renewable surface for the detection of dopamine^20^ and anti-fouling waveforms for serotonin^34^ measurement. Other waveforms include upper limits of 1.4 V or greater in order to detect molecules with higher oxidation potentials such as hydrogen peroxide,^35^ adenosine,^36^ guanosine^37^ and others. Additionally, the surface of the carbon fiber can be modified with polymers for enhanced sensitivity while repealing interferants. By altering the waveform and modifying the surface of CFMEs, scientists have been able to detect and differentiate various neurochemicals apart from dopamine that has led to many exciting applications for both in vivo and ex vivo measurements.

## Carbon electrode modifications for enhanced neurochemical measurements.—

In recent years, numerous assays have been developed to enhance the detection of neurotransmitters with CFMEs. Carbon nanotubes (CNTs), for example, have been utilized due to their high conductivity, aspect (surface area: volume) ratio, which are optimal for neurotransmitter detection.^38^ Discovered by Iijima via arc discharge synthesis,^39–41^ CNTs dipcoated or electrodeposited onto CFMEs have shown enhanced co-detection of dopamine and serotonin with respect to unmodified electrodes.^36^ CNTs functionalized with negatively charged carboxylic acid groups further enhanced detection through an electrostatic interaction, while vertically aligned CNT forests with iron chloride also greatly enhanced sensitivity by over 90%.^42,43^ Fibers and yarns solely made from CNTs also further enhanced neurotransmitter detection.^44^ CNT fiber microelectrodes wetspun from polyethyleneimine (PEI) were found to be fouling resistant to serotonin, while CNT yarn microelectrodes were shown to trap dopamine at the surface.^45–48^ They have a sensitivity independent of the wave application frequency for high temporal measurements of dopamine^49^ and serotonin.^50^ Moreover, carbonaceous material such as CNTs^51^ and carbon nanospikes were grown on metal wires for the development of enhanced neurotransmitter sensors.^52^ Additionally, there are two subtypes of CNTs with single wall- and multi walled CNTS. SW-CNTs are made up of single sheets of graphene rolled into tubes while MW-CNTs are multiple layers of graphene cylinders.^53,54^ Orientation and deposition methods of these CNTs can also impact their sensitivity to neurochemicals.

In addition to carbon nanotubes, CFME have been modified with polymers. Nafion has been utilized by many researchers due to its ability to form cation conducting networks which are able to attract the positively charged DA. The sulfonate group on this polymer repels negatively charged metabolites and interference such as ascorbic acid (AA), which all share similar oxidation peaks.^55,56^ Although, Nafion alone was not stable for reliable measurements, as it did not strongly bond with the carbon fiber surface, PEDOT was utilized.^57^ Electrodeposition of PEDOT:Nafion was accomplished by applying a triangle wave, from +1.5 V to −0.8 V, on a CFME surface submerged in the polymer solution.^58^ Nafion was also found to repel 5-HIAA, a metabolite of serotonin, to enhance 5-HT detection.^59^ PEDOT:Nafion coatings were also shown to increase electron transfer and enhance DA detection,^60^ in vivo,^58^ and in ex vivo zebrafish retina.^61^ However, the two-fold selectivity came at a cost where temporal resolution suffered by 400 ms.^58^ Polyethyleneimine (PEI) coatings were also applied to enhance the detection of anionic neurotransmitters such as DOPAC,^62^ while PEDOT:PEI coatings helped detect and differentiate other dopaminergic metabolites such as DOPAL and 3-methoxytyramine (3-MT).^19,63^ The sensitivity of these different analytes can be characterized by calculating the limit of detection (LOD) measurements. Having a lower LOD indicates the analyte can be detected even when present at nanomolar concentrations. These measurements of common analytes can be found in Table I.

**Figure 2.**
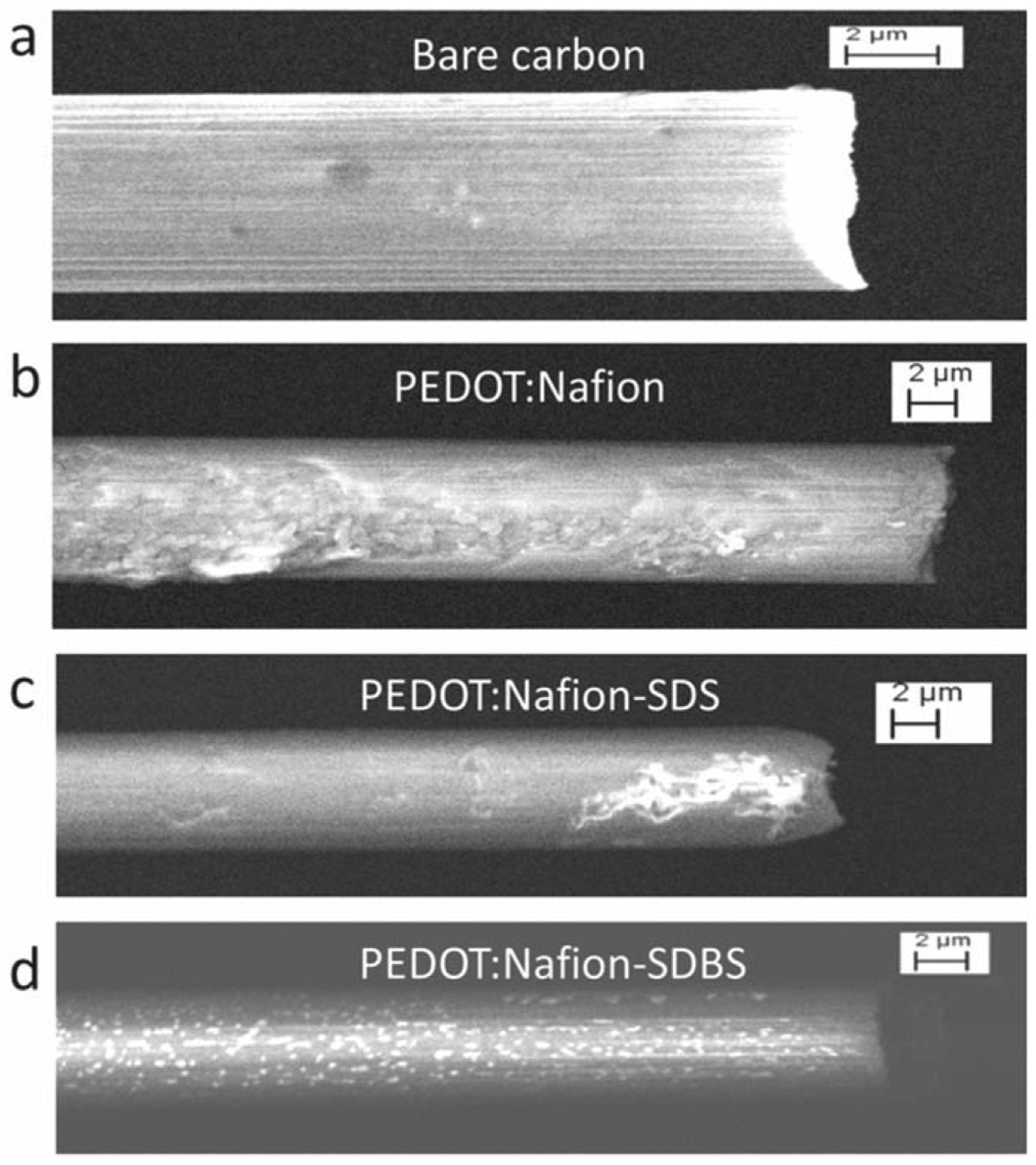
SEM images carbon fiber 7 *μ*m × 100 *μ*m for Bare carbon (a), PEDOT:Nafion (b), PEDOT:Nafion-SDS (c), PEDOT:Nafion-SDBS (d). The PEDOT:Nafion coatings were deposited from a solution containing 200 *μ*M EDOT. Reproduced with permission from Ref. 60. Copyright The Electrochemical Society.

Additionally, surfactants have been used with PEDOT:Nafion electrodes, such as sodium dodecyl sulfate (SDS) and sodium dodecyl benzene sulfonate (SDBS). Scanning electron microscope (SEM) images of these polymers on CFMEs can be seen in Fig. 2. SDS was used as it increased the sensitivity with aiding in Nafion assembly and enhancing conductivity of the PEDOT due to the sulfate group. It was also less sensitive to the anionic compounds such as ascorbic acid. SDBS acted similarly except the improvement was due to the sulfonate group which competed, rather than being incorporated, with Nafion. Both, however, showed greater sensitivity to DA by up to a five-fold increase.^60^ Coating for enhanced detection is not limited to polymers, but can include noble metal-nano particles, specifically gold (Au). Other coatings such as gold nanoparticles, have also enhanced the sensitivity and temporal resolution by increasing the conductivity and electroactive surface area of the carbon electrode substrate.^64^ As gold microelectrodes have been used to detect many NTs and are comparable to CFMEs,^65^ electrodeposition of Au nanoparticles (NP) was considered. Electrodepositing AuNPs can increase the surface area available for NT adsorption as they can be purified and fixed onto the carbon fiber. The electrodeposition was accomplished by electro-reduction of Au^3+^ to Au^0^ and deposition onto the carbon fiber as NPs. Direct comparisons between bare and AuNP CFMEs showed higher sensitivity for dopamine detection. Energy-dispersive X-ray spectroscopy (EDS/EDX) measurements detailed a heterogeneous carbon-gold surface that increased sensitivity and provided for faster electron transfer kinetics.^64^

## Dopamine metabolites.—

Other catecholamines that have been detected using FSCV are metabolites of DA. DOPAL (3,4-Dihydroxyphenylacetaldehyde) is metabolized by monoamine oxidase or MAO and is an aldehyde product of DA. It has been postulated to play an important role in Parkinson’s disease, contributes to reduction of dopamine, and increases toxicity to dopaminergic neurons in the substantia nigra and striatum.^66^ Due to its potential to act as a biomarker for PD, it is necessary to differentiate between DA and DOPAL. Using unmodified CFMEs with the triangle waveform, DOAPL’s oxidation peak was observed at 0.7 V.^17^ When DA and DOPAL were measured together, only a single peak was observed.^63^ To differentiate DOPAL’s peak from dopamine, the electrode surface was modified by the electrodeposition of the polymers polyethyleneimine (PEI) and Poly(3,4-ethyle-nedioxythiophene) polystyrene sulfonate (PEDOT:PSS).^17^ The PEI coated CMFEs had higher sensitivity for DOPAL detection due to the electrodeposited polymer forming a thin, positively charged layer on the electrode’s surface, which attracted the negatively charged DOPAL.^62,63^ Although this enhanced DOPAL detection by repelling the DA molecules, it was not sufficient in detecting two separate peaks for co-detection.

3-methoxytyramine (3-MT) is a post synaptic metabolite of dopamine synthesized via catechol-O-methyltransferase (COMT). Unlike DOPAL, it was easily co-detected and differentiated from DA. 3-MT has markedly slower electron transfer kinetics than dopamine, which has a 0.2 V peak oxidative potential difference from DA.^17^ The co-detection between the two was enhanced upon the electrodeposition of PEI and PEDOT onto the electrode surface, which enhanced adsorption through a heterogeneous electrode surface.

Another pre-synaptic metabolite of DA is DOPAC (3,4-Dihydroxyphenylacetic acid) and is synthesized via MAO. The utility in discerning DOPAC from dopamine lies with its significant role in mitochondrial dysfunction, in tandem with nitric oxide (NO), where oxidative stress and dysfunction can lead to degeneration of dopaminergic neurons.^67^ This catecholamine oxidizes in a quasi-reversible process in a two-electron transfer where only some of the oxidized product is reduced back to its original form. The carboxyl group, which differentiates DOPAC from DA, is deprotonated and gives it an overall negative charge at a physiological pH.^62^ Although both metabolites are detected via the same triangle waveform, DOPAC is also indistinguishable from the DA’s signal without a form of electrode modification. To further increase the selectivity of DOPAC over DA, a novel waveform was used which scanned from 0 V to 1.3 V at 400 V s^−1^. By shifting the holding potential from a negative to neutral voltage, the CFME became more sensitive towards DOPAC due to the diminished electrostatic repulsion of the anion. In combination with that waveform, PEI and Nafion coated CFMEs were used. The PEI coating provided a positive charge to electrostatically attract DOPAC. However, for the co-detection of the two molecules, PEI-CFMEs were used with the DA triangle waveform which produced two distinctive peaks corresponding to DA and DOPAC at approximate 0.6 V and 0.7 V, respectively.^62^

## Norepinephrine.—

Norepinephrine (NE) plays a major role in the central nervous system in arousal, and a mediator of the reward pathway.^68^ NE’s role in stress response has been implicated in multiple disorders related to stress such as addiction and postraumatic stress disorder (PTSD).^69,70^ Clinical studies also indicate NE may play a role in depression.^71^ In animal tissue, NE can be found in the ventral bed nucleus of the stria terminalis (vBNST) via stimulation of the VTA as noradrenergic bundles are seen in this pathway. Dopaminergic neurons do not innervate the fusiform nucleus of this area, making it ideal for detecting NE independently. However, when measured within the BNST, DA and NE yield very similar redox peaks.^72,73^ This is not surprising however, as they have similar structures, are both synthesized from tyrosine, and are only distinguished by a single hydroxyl group.^74^ NT release was also elicited via electrical stimulation of the VTA for NE and the substantia nigra for DA.^75^ In vivo peaks observed on a single CFME produced a wider peak suggesting a combination of other catecholamines or neurochemicals are released when noradrenergic rich areas are stimulated.^76^ To further delineate the signals, simultaneous measurements were taken in the NAc and BNST with two separate CFMEs. The resulting CVs with the triangle waveform were indistinguishable for both analytes. However, to confirm that it was indeed DA and NE being measured in these areas, the pharmacological agents desipramine, an inhibitor of norepinephrine transporter (NET) and yohimbine, an adrenergic receptor antagonist, were used. When administered via intraperitoneal (i.p.) injection in rats, DA exocytosis was unaffected while excess NE remained in the synaptic cleft. This did not affect the NE CV but diminished the DA signal.^73^ Although differentiation was still difficult, these studies indicated that norepinephrine was detectable via CFMEs, further waveform development is still needed.

More recently, a physical surface modification of CFMEs has enhanced not only NE detection but also differentiation from epinephrine (EPI), a neurohormone that plays a role in the fight or flight response.^77^ The diameter of CFMEs were reduced by a wetetching process. The electrode was dipped in as KOH (potassium hydroxide) solution with an applied voltage (+ 7 V) to produce a sharp, conical tip, that facilitated piercing through cellular walls without cellular rupture. Electrochemical conditioning removed the wax coating at the tip, enabling NT detection in cultured adrenal chromaffin cells. A triangle waveform that scanned from +0.1 V to +1.45 V at a scan rate of 800 V s^−1^ was applied to the “nano electrode.” A CV of EPI displayed a secondary peak at the switching potential while NE detection yielded a single peak at approximately 0.65 V.^78^ The results from this work showed promise in differentiating neurochemicals that are structurally similar.

## Hydrogen peroxide.—

Hydrogen peroxide (H_2_O_2_). is another analyte that is involved in; cause cellular level oxidative stress, Also known as an oxygen reactive species (ROS), H_2_O_2_ can form free radicals that that are toxic to surrounding cells, which causes oxidative damage to DNA, carcinogen activation, and tumor promotion.^79,80^ Although lethal effects of DNA repair suppression exist, H_2_O_2_ also plays a role in in redox signaling and cascades in normal functions and neuromodulation.^81^ Enzymatically, it is used as a reporter due to its electroactive properties to indirectly reveal presence of non-electroactive species.^82^ The enzymes (such as glutamate oxidase) convert molecules into reporter molecules which can be oxidized and detected via FSCV, with peak oxidative current corresponding to their relative concentration. This method has been used for other biomolecules such as glucose, lactate, acetylcholine, GABA, and glutamate where ionization is not a possibility.^83–85^

As H_2_O_2_ is a catalyst in electrochemical reactions, it is usually detected with Platinum (Pt) electrode sensors.^86^ Pt electrodes, however, require extensive preparation for proper enzymatic coating, biofoul easily, and lack selectivity.^87^ With slow kinetics and non-reversible oxidation, ideal waveform development for this oxidative species has been limited. Differentiation between H_2_O_2_ and other ROSs is greatly needed for this purpose. For detection, a modified triangle waveform was used with a −0.4 V holding potential, similar to the DA waveform, but with a slightly higher switching potential at 1.4 V. At a scan rate of 400 V s^−1^, a 100 *μ*M bolus of H_2_O_2_ was detected with this waveform.^35^ As the reaction is irreversible, only one peak was seen in the forward scan: an oxidation peak at 1.2 V on the cathodic scan.^35,86^ The signal was pharmacologically validated with the use of catalase, an enzyme commonly used for catalysis of H_2_O_2_ decomposition. This ensured that the signal indeed belonged to H_2_O_2_, and the resulting CV shared the same potential peak. Additionally, by testing other analytes, such as dopamine and ascorbic acid, it was confirmed their oxidation peaks did not interfere with each other using this waveform.^35^

To enhance the selectivity of H_2_O_2_, polymer mPD or 1,3-Phenylenediamine was used with the modified triangle waveform.^88,89^ This created a size-exclusive membrane on the electrode’s surface, preventing other larger molecules from interfering with the H_2_O_2_ signal. The electrodes were electrodeposited for 5 s to ensure other analytes such as adenosine could be successfully filtered out when testing in vivo. In this case, sensitivity was a tradeoff for selectivity where the current had decreased by 30% for the mPD-CFMEs. As H_2_O_2_ is released as a byproduct of cellular respiration, in vivo experiments were performed in the dopamine-rich rat striatum. DA was successfully excluded in the resulting CVs and only an H_2_O_2_ signal was observed. Surface modification also underwent pharmacological confirmation with mercaptosuccinic acid (MCS). MCS was used as an irreversible inhibitor of glutathione peroxidase, an enzyme which reduced H_2_O_2_ to water and in turn, decrease its concentration. It also was redox active with a similar peak as H_2_O_2_ but was successfully filtered out from mPD- CFME detection.^90^ In this instance, both waveform and electrode surface modification achieved differentiation of H_2_O_2_.

## Histamine.—

Histamine is a biogenic monoamine that has been implicated in many functions in the central and peripheral nervous system. It plays roles in consciousness, sleep, allergic responses, and brain disorders such as essential tremors and migraines.^91,92^ Because histamine is electroactive, it can be detected via FSCV, however, it has not been well analyzed. A prior study utilized a triangle waveform that swept from −0.4 V to 1.4 V, where an oxidation peak at 1.3 V was observed on the backward scan and a reduction peak at −0.2 V.^93^ A broad peak was also noted between 0.5 V and 0.8 V, which was determined to be an auxiliary peak. The switching potential was capped at 1.4 V to avoid overlap with other analytes, such as adenosine, although it could not be co-detected otherwise.^94^ Moreover, switching potentials are generally capped at 1.45 V to avoid water electrolysis in the buffer solution.

The electrochemical oxidation scheme of histamine is not fully known and oxidative peaks that are seen in the CV might be due to non-faradaic processes. This can be attributed to the adsorption of histamine affecting the electrical bilayer on the electrode at commonly used switching potentials. To bypass the charging and discharging currents, Samaranayake et al. developed a novel waveform to enhance the selectivity and detect histamine before the switching potential and on the forward scan.^95^ Denoted the histamine selective waveform (HSW), they scanned from −0.7 V to 1.1 V, while resting at −0.7 V at a scan rate of 600 V/sec. This yielded the strongest peak and successfully excluded H_2_O_2_ and adenosine. To ensure selectivity of histamine, codetection was performed with the electrochemically similar DA, 5-HT, and adenosine. The resulting peak for histamine was seen at 0.3 V, and distinct from DA, 5-HT, and adenosine, with oxidative peaks seen at the switching potential.^95^

Slight alterations to this HSW were needed when measurements were made in mice brain tissue. The nuclei of the posterior hypothalamus were targeted as it contained a dense population of histamine cell bodies. These nuclei, such as the premammillary nucleus (PM) and tuberomammillary nucleus (TMn), have afferent neurons that connect to the forebrain via the medial forebrain bundle (MFB).^96^ When the MFB was stimulated, histamine was detected in the PM.^89^ With the resting potential changed to −0.5 V, the histamine signal was verified in vivo with limited electrode fouling, which was not seen at a resting potential of 0.3 V.^95^ Finally, they confirmed this signal with tacrine, an inhibitor of histamine metabolism via *N*-methyltransferase (HNMT).^97^ Tacrine significantly increased the reuptake time of histamine. Similar results were seen when a more specific agent was used, the histamine (H3) antagonist thioperamide.^95,98^ The drug agents raised the cytosolic concentration of histamine, increased the clearing time, and amplified the signal electrochemically.

Histamine was also measured by the Venton group with the sweep ranging from −0.4 V to 1.3 V or 1.45 V at a scan rate of 400 V s^−1^. Histamine was proposed to undergo a single electron transfer oxidation on an imidazole nitrogen resulting in a radical. The primary peak was observed at approximately 1.2 V. and smaller secondary peak at.8 V on the forward scan was attributed to the electropolymerization of that radical. The radical also contributed to electrode fouling and caused a decrease in current overtime compared to the initial reading.^93^ These studies indicate that better understanding electron transfer schemes can aid in that developing a single ideal waveform but is not always necessary to visualize biomolecules. Additionally, waveform modification can exclude interferents and show a single peak, but further work is needed for co-detection to manifest several individual peaks for each analyte.

## Serotonin.—

Serotonin (5-HT) plays many roles in the brain such as in depression, mood, emotion. Microdialysis with HPLC has been used for 5-HT detection with a response time as quick as under a minute.^51^ However, to achieve a higher temporal resolution, FSCV with waveform modification has been utilized to isolate and detect 5-HT. Previously, with the HSW for histamine detection, 5-HT’s peak oxidative current overlapped with DA.^89^

To better visualize 5-HT alone, FSCV assays utilized waveforms that used a high scan rate to avoid the adsorption of unwanted products with a piecewise function, known as the Jackson waveform (JWF).^34^ Since its conception, it has been modified with two additional waveforms to detect 5-HT at both 1000 V s^−1^ and 400 V s^−1^. Serotonin fouls on the surface of the electrode at higher concentrations and timescales and diminishes sensitivity. An extended serotonin waveform (ESW) was developed such prevent fouling at the electrode surface. Similar to the JWF, this waveform used a piecewise function from 0.2 V to 1.3 V then −0.1 V to 0.2 V, at 1000 V s^−1^. As higher switching potentials were used to renew the CFME surface and remove impurities,^18^ this higher scan rate was paired with a modified piecewise function. ESW yielded an oxidation peak at 0.9 V, similar to JWF but more resolved, and a reduction peak at 0.0 V.^99^

**Figure 3.**
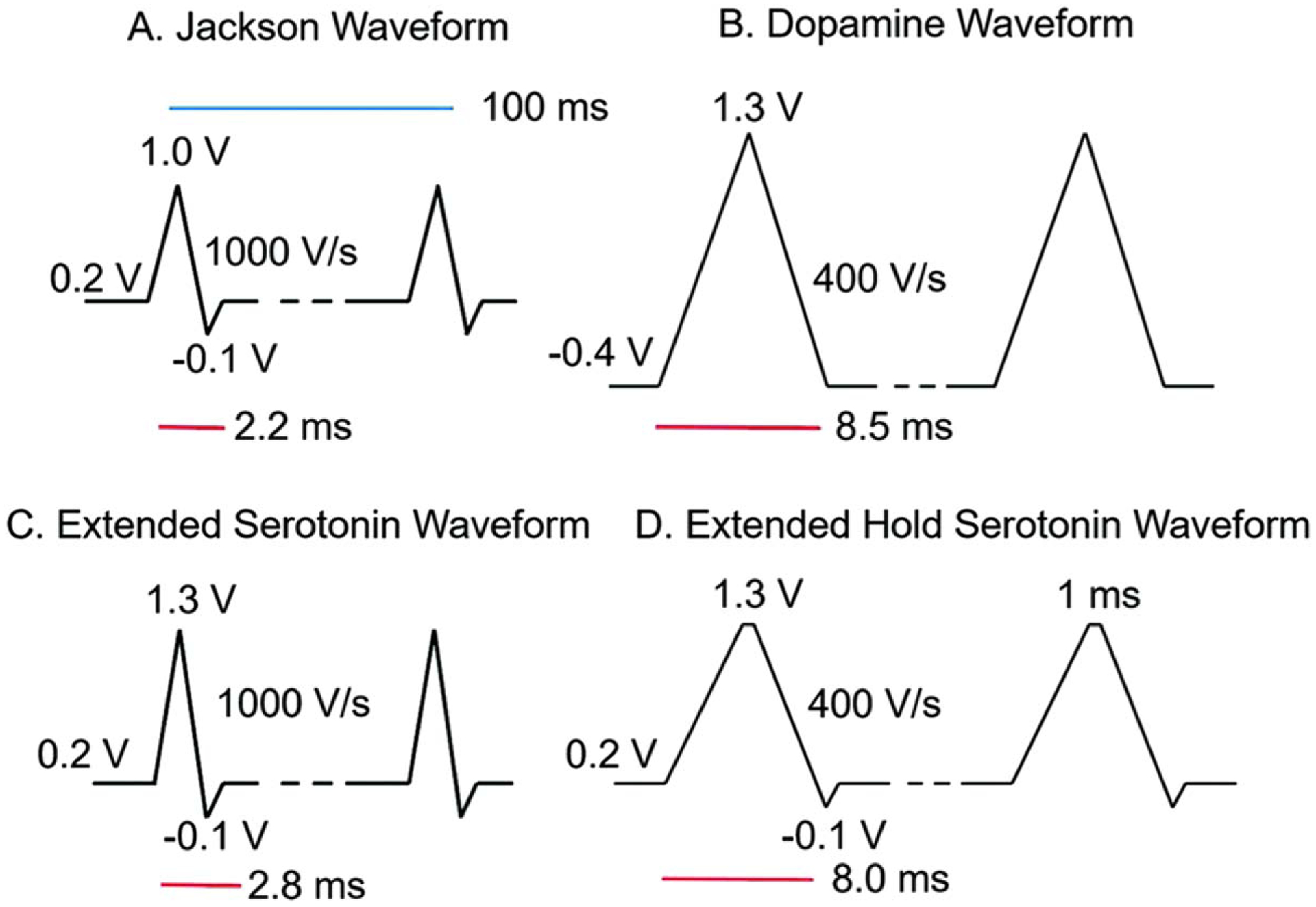
Waveforms tested. (A). Traditional serotonin “Jackson” waveform with a 1.0 V switching potential and 1000 V s−1 scan rate. (B). Traditional dopamine waveform with a −0.4 V holding potential, extended 1.3 V switching potential, and 400 V s−1 scan rate. C. Extended serotonin waveform (ESW) with 1.3 V switching potential and 1000 V s−1 scan rate. (D). Extended hold serotonin waveform (EHSW) with a 1 ms hold at 1.3 V and 400 V s−1 scan rate. All waveforms were repeated at 10 Hz. Reproduced from Ref. 99 with permission from The Royal Society of Chemistry. K. E. Dunham and B. J. Venton, *Analyst*, ***145***, 7437–7446 (2020).

Additionally, another waveform was established that made use of a lower scan rate. The extended hold serotonin waveform (EHSW) was comparable the ESW except the switching potential of 1.3 V was held for 1 ms, and at a scan rate of 400 V s^−1^. The “sawhorse” shape aided in 5-HT detection as holding at the switching potential allowed extra time to oxidize the surface of the electrode. The EHSW yielded similar results to the DA triangle waveform for 5-HT with redox peaks at 0.0 V and 0.6 V, respectively.^99^ The different waveform shapes are shown in Fig. 3. In comparing the 5-HT waveforms, it was found that the JWF fouled the most, minimal fouling occurred with both 5-HT waveforms, and no fouling occurred with the DA waveform. This was not surprising as the DA waveform is the most anti-fouling due to its ability to renew the electrode surface.^18^ The fouling was attributed to a downstream metabolite, 5-hydroxyindoleacetic acid (5-HIAA), which produced a free radical that fouled the surface, as seen with histamine. This radical intermediate dimerized and electropolymerized and, thus, fouled the CFME surface.

To determine the effect of 5-HIAA fouling, CFMEs were soaked in 1 *μ*M 5-HIAA for an hour with an applied waveform and compared to an hour-long soak in 5-HT and phosphate buffered saline (PBS) for a control. CVs before and after the soak showed that the JWF displayed the greatest amount of fouling. These experiments suggested switching potentials above 1.3 V renewed the surface and removed electropolymerized films. While the JWF attempted to accomplish this with an increased scan rate, both modified 5-HT waveforms outperformed the JWF. The dopamine waveform was surprisingly more sensitive for 5-HT than DA, and the negative holding potential eliminated fouling almost entirely. However, it was not specific for serotonin over dopamine and detected both analytes at near equal ratios. Comparing the four waveforms, the JWF was the most selective for 5-HT but was not ideal due to electrode fouling. ESW displayed enhanced sensitivity and selectivity, while the EHSW and DA waveforms had greater sensitivity. The ESW was the better choice for differentiating between 5-HT and DA.^99^ This showed the need for waveforms that not only have high selectivity for the choice analytes but have tailored anti-fouling properties for long term use of an electrode.

In addition to waveform development, electrode modification was also performed with Nafion coating to increase the selectivity of the CFME to 5-HT over 5-HIAA in vivo.^59^ As previously stated, Nafion provides a greater sensitivity to cations such as 5-HT, over anions such as 5-HIAA which retains a negative charge in solution.^100^ Hashemi et al. made a modification to previously used Nafion electrodeposition protocols to ensure a thin and uniform coating on the CFME.^59,101^ This Nafion-CFME, in tandem with the JWF, was inserted into the rat substantia nigra reticulata (SNR) and the dorsal raphe nucleus (DNR) was stimulated. Low frequency stimulation, under 60 Hz, evoked a corresponding concentration of 12.7 nM of 5-HT in the SNR. These were similar concentrations of extracellular 5-HT previously detected by HPLC.^59,89,102^ Lastly, pharmaceutical agents were used to ensure the signal seen in vivo belonged to 5-HT. DA interferants were not present as dopaminergic neurons do not project from the DNR to the SNR. Dopamine transporter (DAT) inhibitor; GBR 12909, and serotonin transporter (SERT) inhibitor; escitalopram, were administered to rats to inhibit the reuptake of the two NTs. The resulting CVs revealed that inhibiting DAT did not alter the signal while escitalopram significantly increased the released concentration of 5-HT and a near four-fold increase in clearance time (t_1/2_).^59^

The combination of the above experiments validated the detection of 5-HT in vivo for the first time with the Nafion-CFME and the JWF. Although multiple waveforms were presented, they all were able to detect 5-HT indicating that there are several ways to identify the same analyte with FSCV.

## Adenosine.—

Adenosine is a neuromodulator found in the brain that can have neuroprotective effects in response to hypoxia and neurodegenerative diseases.^103^ Adenosine is difficult to differentiate from biomolecules that share similar oxidation peaks, such as H_2_O_2_, ATP, and histamine. Specifically, with ATP and H_2_O_2_, oxidation peaks were nearly identical, at 1.45 V, when detected with a triangle waveform that scanned from −0.4 to 1.45 V at 400 V sec^−1^.^104^ To enhance selectivity, a modified sawhorse waveform was developed which scanned from −0.4 V to 1.35 V, held for 1.0 ms, then back to −0.4 V, at 400 V s^−1^. The hold at the switching potential enabled more adenosine molecules to oxidize on the electrode’s surface, similar to 5-HT. This holding time was set to 1.0 ms as a lower time (0.5 ms) resulted in less current, and a 1.5 ms holding time caused a greater background current instability. The sawhorse waveform also displayed a drop in capacitive current where the voltage was held, which caused a decrease in faradaic currents. For species that are adsorption controlled, the current returns to zero once the species is fully oxidized. The current decay is slower in species that are diffusion controlled, such as hydrogen peroxide, where the current continues to decay at the holding potential.^104^ Adenosine, and ATP, fall into the latter category and are diffusion controlled with current falling at the switching potential. With the sawhorse waveform, a primary peak for adenosine was observed at 1.45 V and a smaller secondary peak, especially at higher concentrations, at 1.0 V. This secondary peak was attributed to a change in background current in response to adenosine adsorption on the electrode’s surface. With electrode surface renewal, it is possible that the 1 ms holding time was insufficient for full restoration, resulting in the extra adsorption peak.^104^ Additionally, the oxidation of adenine, the nucleobase of adenosine, also had a secondary peak but with at a lower current. Due to this adsorption peak being present for adenosine, adenine, and ATP, but not H_2_O_2_, it may be a result of the product of the nucleobase.^104^

In order to illustrate the versatility of the adenosine sawhorse waveform, electrically stimulated measurements were performed in rat brain slices. Using the same sawhorse waveform, mechanically stimulated adenosine release was measured.^105^ In vivo CVs were similar to the exogenous applied adenosine, apart from an extra negative peak seen at the beginning of the CV, which may have been artifacts from electrode insertion into the brain. This showed that the sawhorse waveform was sufficient in identifying adenosine among interferants both in vitro and in vivo.^104^

**Figure 4.**
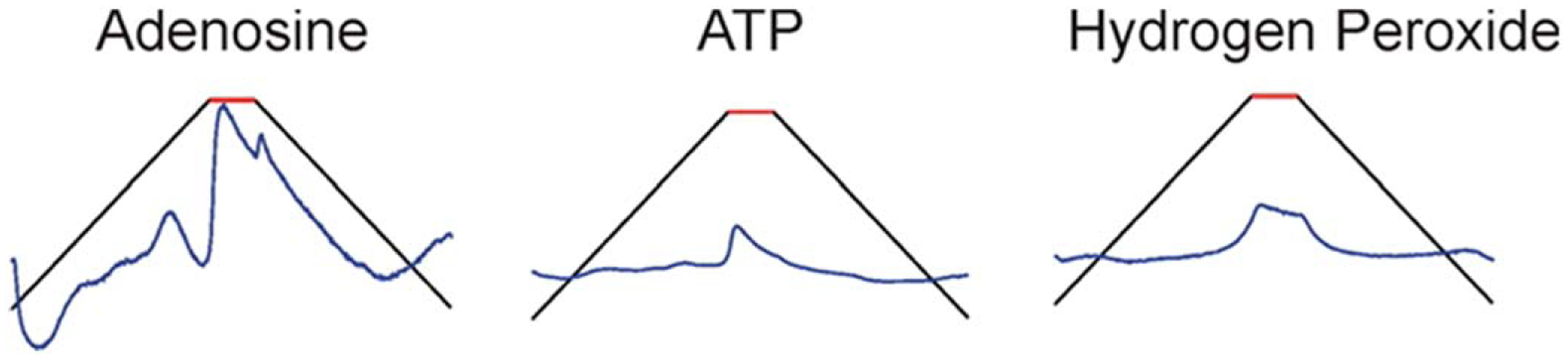
A schematic showing background subtracted, unfolded CVs of adenosine, ATP, and hydrogen peroxide using the modified sawhorse waveform.^104^ Copyright 2014 American Chemical Society.

The modified sawhorse waveform enabled discrimination between adenosine and similar oxidative peak biomolecules, however, it was not possible to discriminate a mixture of ATP and adenosine. Although, individual detection was possible as the resulting CVs did not share identical signals (Fig. 4). In adenosine’s case, electrode surface modification did not greatly change the results. Nafion-CNT electrodes were 6 times more sensitive to adenosine over ATP due to the negative charge of the phosphate (PO_4_^3−^), but the CV shapes were not significantly different.^57^

## Guanosine.—

Guanosine (Gn) is another important purine neuromodulator that not only impacts adenosine levels but is structurally similar to adenosine. The signaling mechanism for Gn is not well understood but both nucleosides are found in astrocytes and are the main extracellular source of purines. Additionally, both can control glutamate transmission, implicating a joint role in Alzheimer’s Disease (AD).^106^ This guanine-based purine is also known to increase in concentration in response to brain injury. To pick out Gn from adenosine, the initial guanosine waveform scanned from −0.4 V to 1.3 V, at a rate of 400 V s^−1^ and produced only two oxidation peaks. The primary oxidation peak was observed at the switching potential, 1.3 V, on the backward scan. If the switching potential was increased to 1.45 V, the primary peak shifted to the forward scan, indicating slow kinetics of guanosine. A secondary peak was observed at 0.8 V on the forward scan. The presence of two peaks indicated the oxidation reaction involved a two-electron transfer on the guanine moiety. First, a radical formed and dimerized prior to detection. The second, although reversible, was highly favored. As the original waveform did not exceed 1.3 V, an adenosine signal was successfully omitted. The waveform and assay were successfully utilized to detect exogenously applied guanosine in an ex vivo rat brain slices.^37^ However, due to their shared roles in the nervous system, it is important to be able to detect both, simultaneously.^106^

**Figure 5.**
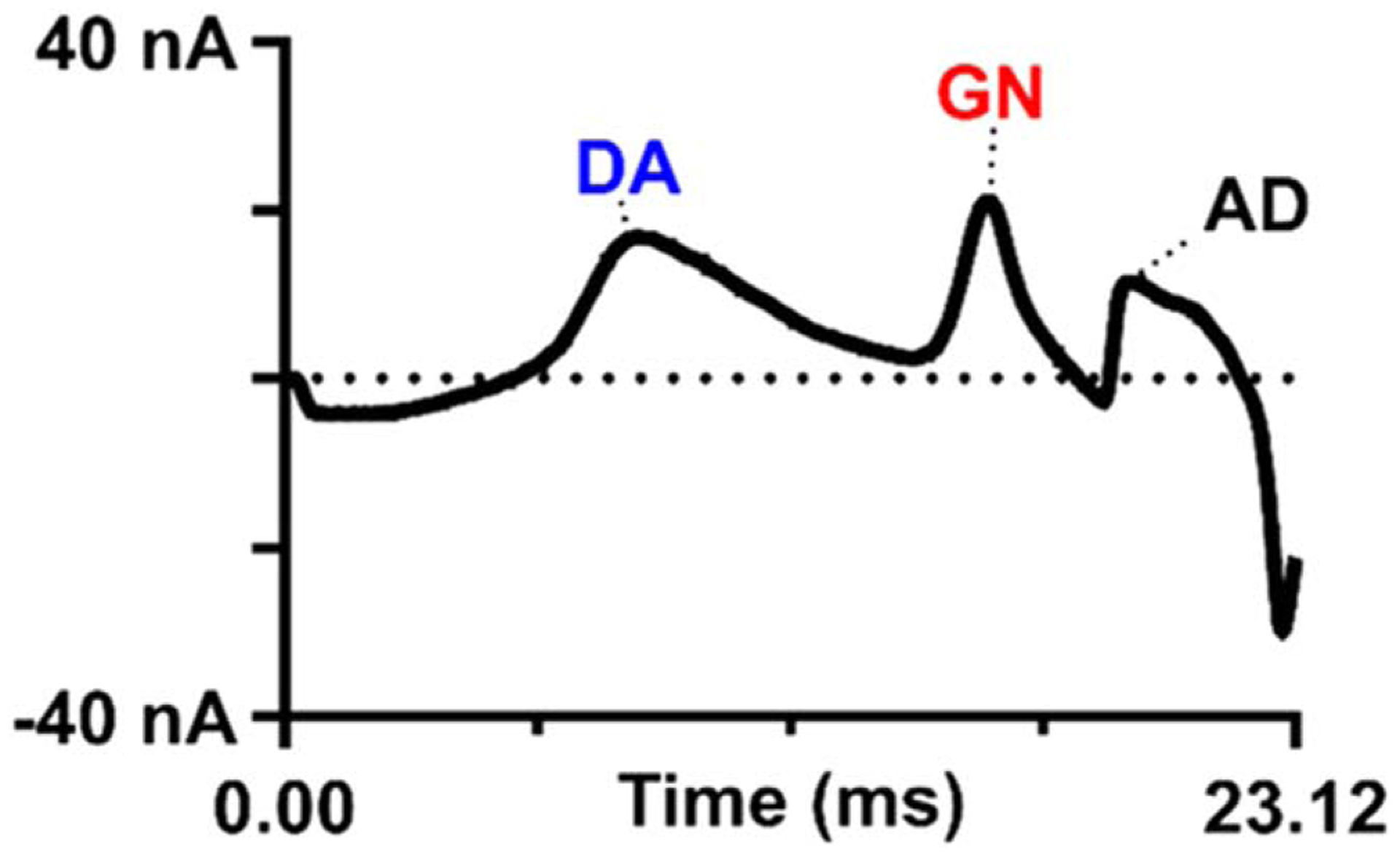
Triple detection of guanosine, adenosine, and dopamine using the scalene waveform. (A) Example false-color plot showing 5 *μ*M (each) dopamine (DA), guanosine (GN), and adenosine (AD). Dopamine oxidation occurs at 0.46 V when the modified waveform is used. (B) Opened cyclic voltammogram for the purine–dopamine mix. The Δt between dopamine and guanosine, its nearest neighbor, is 6.96 ms.^105^ Reprinted with permission from M. T. Cryan and A. E. Ross, *Anal. Chem*., ***91***, 5987–5993 (2019). Copyright 2019 American Chemical Society.

To optimize a waveform that would detect both guanosine and adenosine, a modified scalene waveform was used with a slower rise to the switching potential from −0.4 V to 1.45 V, at 100 V s^−1^, then faster at 400 V s^−1^ on the backward scan down to −0.4 V. With the scalene waveform, guanosine’s primary peak was observed at 1.2 V and the secondary peak was observed at.66 V. Adenosine’s primary peak appeared at 1.29 V, with the secondary peak at 0.88 V. The slower waveform enhanced the separation between the primary and secondary peaks of both purines. The distance between the peaks also indicated that adenosine’s electron transfer was slower than that of guanosine. Also, the primary oxidation steps for both were irreversible and resulted in peak potential shifts as a function of scan rate. This novel scalene waveform yielded a nanomolar limit of detection (Table I) and resolved all four peaks within an unfolded single CV. When utilizing the guanosine triangle waveform, on the other hand, there was minimal separation of peaks and secondary peaks for both purines were absent.^105^ Analyte multiplexing was also performed with the addition of DA (Fig. 5). All three analytes had distinct peaks with DA oxidation seen at 0.46 V, however, secondary oxidation peaks for either adenosine ad Gn were not present. Although co-detection for all three analytes has yet to be validated in vivo, Gn and adenosine primary oxidation peaks have been confirmed in rat caudate putamen slices.^105^

## Melatonin.—

In addition to nucleosides, recently, the hormone melatonin has also been measured with CFMEs and FSCV. Melatonin regulates circadian rhythms, body temperature, oxidative stress, and mitochondrial homeostasis.^106–108^ Early research limited melatonin production to the pineal gland, but newer literature shows melatonin receptors and synthesis in the retina, lymphocytes, and gastrointestinal tract.^109^ Immune related functions also include modulation of inflammation where melatonin may suppress inflammation via COX-2 enzymes.^110^ With such critical roles in the nervous system, a specific waveform was developed to detect and enhance sensitivity of melatonin with CFMEs.

The oxidation scheme of melatonin involves a single electron abstraction which generates a radical cation. This is further oxidized into a quinoneimine by electron and proton loss. This oxidation is irreversible and is adsorption controlled. Because the quinoneimine is highly reactive, it electropolymerizes in solution and leads to unwanted adsorption products, causing it to foul the surface of the electrode.^111^ Using the traditional triangle waveform, melatonin readily fouled the electrode, as seen with 5-HT, which decreased sensitivity and gave rise to a secondary peak due to the radical byproduct. During in vitro experiments, the secondary product lingered on the electrode’s surface. To circumvent this, a modified waveform was developed, which scanned from 0.2 V to 1.3 V at 600 V s^−1^ and the higher scan rate eliminated the radical peak. To ensure selectivity, this waveform was tested with structurally similar serotonin, dopamine, and their respective metabolites; 5-HIAA, 6-hydroxymelatonin (6-HMA), and N-acetyl serotonin (NA-5HT). As the modified waveform lacked a negative holding potential, DA was not measured with this assay. 5-HT had a higher sensitivity, but a different oxidation peak potential than melatonin, which was observed at 1.1 V, allowing for peak distinction. Melatonin and 5-HT were then co-detected and serotonin’s oxidation peak was observed at.7 V. However, it was more challenging to differentiate from serotonin’s downstream metabolite, NA-5HT and melatonin’s metabolite, 6-HMA.^112^

To study melatonin in animal tissue, mesenteric lymph nodes (MLN) were excised from female mice. This was the first instance of melatonin detection in a live lymph node and proper intact sampling was necessary as the structure and state of the tissue could impact the signal. MLNs play a critical role in immune system relaying in the gastrointestinal wall and may serve as another source of melatonin in the gut.^113^ In a similar fashion as previous animal studies, melatonin was exogenously applied near the inserted CFME in the MLN. The resulting CV shape was similar to the in vitro signal, which illustrated applicability for using these assays in biological tissue.^112^ Moreover, recent studies have also measured exogenously applied melatonin in brain tissue using square wave voltammetry as FSCV is deemed too fast to monitor slow changes in concentration.^114^

## Neuropeptides.—

Using CFMEs with FSCV for detecting biomolecules is not limited to small molecules. Recent assays include the detection neuropeptides such as enkephalin as well. Originally, this was challenging as neuropeptides are made up of multiple amino acid chains with limited oxidizable residues. The larger size may prevent adsorption onto the electrode’s surface, and the ionized amino acid may be difficult to discern on the CV when multiple oxidizing amino acids are present. However, certain smaller peptides have been detected with voltammetry through the oxidation of the ionizable tyrosine or methionine.^115,116^ Moreover, other peptides such as insulin^117,118^ and glucagon^119^ have been measured with amperometry. The ability to detect neuropeptides with FSCV has expanded the potential applications where a carbon fiber is able to detect large and complex molecules as opposed to solely small molecules.

Enkephalin, or ENK, is an opioid neuropeptide involved in a wide variety of functions, including analgesic effects, cell proliferation activation, and can act on various, nonspecific opioid receptors.^120^ There are two different types of ENK that arise from proteolytic cleaving: methionine-enkephalin (M-ENK) and leucine-enkephalin (L-ENK).^121,122^ The detection of ENKs, however, becomes difficult due to the presence of tyrosine which requires higher oxidation potentials and fouls the electrode surface. To overcome these challenges, the modified sawhorse waveform (MSW) was designed and used to detect M-ENK.^123^ The MSW utilized two distinct san rates in each anodic sweep with a holding potential at −0.2 V that ramped up to 0.6 V at 100 V s^−1^. The potential was increased to 1.2 V at 400 V s^−1^ and held for 3 ms before returning to −0.2 V, also at 100 V s^−1^. The faster scan rate was used around the oxidation peak to capture tyrosine’s oxidation, while the slower scan rate at the beginning and end of the MSW reduced faradaic signals from interfering analytes. The resulting CV had two distinct peaks for M-ENK: at 1.0 V corresponding to tyrosine and at 1.2 V for methionine. The other amino acids in the neuropeptide were not electroactive. On a conventional triangle waveform, M-ENK did produce multiple peaks, but surface fouling readily occurred. Additionally, with multiple non-selective peaks, the triangle waveform was less than optimal, hence, the need for the modified waveform. The selectivity of the MSW was also tested with dopamine to ensure interfering signals were not present. At the DA triangle waveform, a single wide peak was observed when a mixture of 0.5 *μ*m M-ENK, 1 *μ*M DA, 10 *μ*M ascorbic acid, and a + 0.1 pH shift was tested. At the MSW, dopamine retained its own peak around.6 V with a wider peak near the switching potential attributed to M-ENK. When tested with L-ENK, the methionine peak was not present, but the tyrosine peak was observed.^123^

Waveform validation ex vivo was performed in adrenal gland slices of male Sprague-Dawley rats. The adrenal gland was chosen due to this region containing high concentrations of proenkephalin peptides in addition to secreting catecholamines.^124,125^ These properties of the chromaffin cells, found in the medulla of adrenal glands, made them ideal to test the MSW. First, an in vitro mixture of M-ENK and NE, the catecholamine in question, was tested with the new waveform. The adrenal slices were then electrically stimulated, and the resulting CVs compared. Unfolded CVs from the in vitro flow cell and ex vivo tissue data showed matching oxidation peaks. The data suggested the MSW successfully detected M-ENK and may facilitate the detection of other tyrosine containing opioid neuropeptides.^123^

## Conclusions

FSCV is not the only technique used to detect biomolecules, but the high spatial and temporal resolution, easy equipment setup, and ability to manually produce and modify CFMEs, has made this technique a standard in analytical chemistry and neuroscience research. Over the years, this method has been utilized for the detection of dopamine, which is important for understanding the basis of many neurological diseases, behaviors, learning and memory, and drug abuse among others. For other neurotransmitters, our understanding of their physiological roles in vivo has previously been limited by our ability to detect and distinguish them from one another. However, more recently, assays have been developed to not only isolate their signal, but detect additional biomolecules such as neurohormones, neuropeptides, and DNA bases.^126–128^ With the help of surface polymer coatings of CFMEs and waveform modifications, it has become more facile to identify multiple analytes in vitro, ex vivo, and in vivo. With novel sensors, the possibility of clinical relevance and human patient work may also be a possibility.^94,129^ Enhancing the detection of neurochemicals will further help in understand their complex roles in vivo. We have summarized the recent advancements made in FSCV as formulating better sensors with high spatiotemporal resolution is a critical step in understanding the chemical processes and neuroanatomy of specific brain regions.

## Figures and Tables

**Table I.** Table of analytes discussed. Name, acronym, the type of CFME used for detection, the limit of detection (LOD), type of buffer solution used, and corresponding waveform used for detection.

Analyte	Acronym	Method of Detection	LOD (*μ*M)/Sensitivity	Buffer	Waveform
Dopamine^60^	DA	PEDOT:Nafion-SDBS CFME	9 nM	Tris Buffer	Triangle
3,4-Dihydroxyphenylacetaldehyde^63^	DOPAL	PEDOT-PEI CFME	100 nM	aCSF	Modified
					Triangle
3,4 dihydroxyphenyl acetic acid^60^	DOPAC	PEDOT:Nafion-SDS CFME	19 nM	Tris Buffer	Modified
					Triangle
Norepinephrine^75^	NE	CFME	4 *μ*M	Tris Buffer	Triangle
Hydrogen Peroxide^104^	H_2_O_2_	CFME	50 *μ*M	Tris Buffer	Modified
					Triangle
Serotonin^99^	5-HT	CFME	.6 nM	PBS	Piecewise
Histamine^95^	His	CFME	1 *μ*M	Tris Buffer	HSW
Adenosine^104^	Adn	Nafion-CNT CFME	7 nM	Tris Buffer	Modified Sawhorse
Guanosine^105^	Gn	CFME	50 nM	Tris Buffer	Scalene
Melatonin^112^	ME	CFME	24 nM	Tris Buffer	Triangle
Enkephalin^120^	ENK	CFME	.5 *μ*M	PBS	MSW
